# Association of Patient-reported Outcomes With Clinical Outcomes After Distal Humerus Fracture Treatment

**DOI:** 10.5435/JAAOSGlobal-D-19-00122

**Published:** 2020-02-06

**Authors:** Abhiram R. Bhashyam, Yassine Ochen, Quirine M.J. van der Vliet, Luke P.H. Leenen, Falco Hietbrink, Roderick M. Houwert, George S.M. Dyer, Marilyn Heng

**Affiliations:** From the Harvard Combined Orthopaedics Residency Program, Boston, MA (Dr. Bhashyam); the Department of Surgery, University Medical Centre Utrecht, Utrecht, The Netherlands (Dr. Ochen, Dr. van der Vliet, Dr. Leenen, Dr. Hietbrink, and Dr. Houwert); the Department of Orthopaedics, Brigham and Women's Hospital, Harvard Orthopaedic Trauma Initiative, Harvard Medical School (Dr. Dyer); and the Department of Orthopaedics, Massachusetts General Hospital, Harvard Orthopaedic Trauma Initiative, Harvard Medical School (Dr. Heng), Boston, MA.

## Abstract

In this study, we assessed the patient-reported outcomes of distal humerus fracture treatment using Patient-Reported Outcomes Measurement Information System (PROMIS) or QuickDASH (Disabilities of the Arm, Shoulder, and Hand) scores and the association between patient-reported outcomes and clinical outcomes.

**Methods:**

We performed a retrospective cohort study of 76 adult patients who sustained an acute distal humerus fracture between 2016 and 2018; 53 patients completed at least one patient-reported outcome measure used to assess physical function (PF) during their routine follow-up care (69.7% response rate). The average time to follow-up patient-reported outcome measure was 10.3 months. Patients completed the PROMIS PF 10a, PROMIS upper extremity (UE) 16a, and/or QuickDASH based on the treating institution/service. In addition, the PROMIS Global (Mental) subscale score was used as a measure of self-rated mental health. To assess clinical outcomes, we measured radiographic union, range of motion, and postoperative complications.

**Results:**

Most fractures were intra-articular (67.9%), and 84.9% were treated surgically. After treatment, 98.1% of fractures united radiographically. By the final follow-up, the average arc of motion was 18° to 122°. Average (±SD) PROMIS PF and UE scores were 41.7 ± 11.1 and 40.8 ± 12.4, respectively. The average QuickDASH score was 39.4 ± 26.5. The arc of flexion-extension and PROMIS Global (Mental) score were independently associated with PROMIS PF and PROMIS UE scores.

**Conclusions:**

We found that clinical factors (the arc of flexion-extension) and patient psychological factors (PROMIS Global [Mental] score) were independently associated with PROMIS measures of PF after distal humerus fracture treatment. These data can be used to contextualize patient outcomes and guide patient expectations.

Fractures of the distal humerus account for 2% of fractures in the adult population (approximately 30% of all humeral fractures).^1-3^ An increase in the annual incidence of distal humeral fractures has been reported, likely because of a growing older population.^4,5^ In general, these injuries are treated surgically with open reduction and internal fixation (ORIF), but some patients may still be managed with nonsurgical treatment.^1^

Although several studies have evaluated clinical outcomes of distal humeral fractures, fewer studies have explored the association between clinical and patient-reported outcomes (PROs).^6,7^ A recent systematic review identified 109 articles assessing the outcomes of acute distal humeral fracture but found that clinical and PROs were not consistently reported, making accurate comparison of treatment effectiveness difficult.^8^ In addition, the review found that general health surveys were rarely reported and comparison using Patient-Reported Outcomes Measurement Information System (PROMIS) instruments were not possible.^8^

PROMIS instruments are increasingly used to evaluate PRO for upper extremity (UE) injuries because they can be administered and scored in a standardized manner, allowing for quality assessment across medical and surgical fields.^9,10^ In addition, several studies have demonstrated that PROMIS scores correlate with legacy instruments used to measure the PRO of orthopaedic UE trauma patients.^11-13^ Few studies have assessed if there is an association between PRO (eg, PROMIS instruments, QuickDASH) and clinical outcomes.^14,15^ We hypothesized that the variation in PROMIS scores is associated with clinical outcomes. Therefore, in this study, we collected PRO after distal humerus fracture treatment using PROMIS or QuickDASH scores and then explored the association between PRO and clinical outcomes.

## Methods

### Study Design

This study was approved by our institutional review board. We performed a retrospective cohort study of 85 consecutive adult patients (>18 years old) who received treatment at one of two American College of Surgeons Level 1 Trauma Centers from January 2016 to February 2018 for an acute distal humerus fracture. Starting in January 2016, collection of patient-reported outcome measures (PROMs) was standardized in the orthopaedic clinics at both hospitals. Patients were excluded if their injury was initially treated at an outside hospital or if they had a pathologic or periprosthetic fracture. Patients who had zero follow-up visits (five patients) or were in hospice care (one patient) were also excluded, as were patients treated with total elbow arthroplasty (three patients). From the 76 eligible patients, 53 patients completed at least one follow-up PROM used to assess physical function (PF)/UE disability (69.7% response rate) with an average follow-up of 10.3 months (Table 1).

**Table 1:** Sociodemographic and Clinical Characteristics of Responders Versus Nonresponders

	Responders	Nonresponders	
N	53	23	
Percent of total patients (N = 76)	69.7	30.3	

	Responders	Nonresponders	*P* Value
	No. of Patients (%) or Mean ± SD	No. of Patients (%) or Mean ± SD	
Sociodemographic characteristics			
Age at injury (yr)	54.5 ± 20.4	65.1 ± 19.4	**0.038**
Male	17 (32.1)	8 (34.5)	0.509
Caucasian	44 (83.0)	15 (65.2)	0.081
Median income ($)^b^	93,600 ± 30,600	83,600 ± 30,900	0.200
Marital status			0.15
Single	25 (48.1)	5 (23.8)	
Married	22 (42.3)	11 (52.4)	
Widowed	4 (7.7)	3 (14.3)	
Divorced	1 (1.9)	2 (9.5)	
Insurance type			**0.025**
Private	30 (56.6)	6 (26.1)	
Medicaid	3 (5.7)	5 (21.7)	
Medicare	20 (37.7)	12 (52.2)	
Injury-related characteristics			
HET	7 (13.2)	3 (13.0)	0.648
Open fracture	3 (5.7)	4 (17.4)	0.119
Multiple injuries	9 (17.0)	3 (13.0)	0.477
AO/OTA fracture classification			0.550
A (extra-articular)	17 (32.1)	10 (43.5)	
B (partial articular)	12 (22.6)	53 (13.0)	
C (complete articular)	24 (45.3)	10 (43.5)	
Procedure-related characteristics			
Procedure			0.133
Closed treatment	8 (15.1)	3 (13.0)	
ORIF	22 (41.5)	16 (69.6)	
ORIF + subcutaneous ulnar nerve transposition	8 (15.1)	2 (8.7)	
ORIF + submuscular ulnar nerve transposition	15 (28.3)	2 (8.7)	
UE specialist	24 (45.3)	9 (39.1)	0.405
Inpatient surgery	31 (58.5)	15 (65.2)	0.387
Post-procedure characteristics			
Length of stay (d)	2.3 ± 2.3	2.3 ± 1.8	0.933
Discharge to rehab	6 (11.3)	5 (21.7)	0.200
Follow-up time (mo)	10.3 ± 7.1	5.8 ± 4.2	**0.001**

DASH = Disabilities of the Arm, Shoulder, and Hand, HET = high-energy trauma, OTA = Orthopaedic Trauma Association, OR = odds ratio, ORIF = open reduction and internal fixation, UE = upper extremity

b Median income from ZIP code of residence based on 2016 census data.

Boldface indicates statistical significance.

### Patient-reported Outcome Measures

Patients completed the PROMIS PF 10a, PROMIS UE 16a, and/or the QuickDASH to assess PF and UE disability on a tablet device as part of their routine follow-up visit at the treating institution.^9,11,13,16,17^ In addition, the PROMIS Global was completed and the PROMIS Global (Mental) subscale score was used as a measure of self-rate mental health.^18^ The PROMIS instrument scores range from 0 to 100 with a mean score of 50 for the general population of the United States (SD of 10).^9^ The QuickDASH is an 11-item questionnaire that measures UE-specific disability with higher scores reflecting more severe disability (range of 0 to 100) and a mean of 11 points reflecting the general US population average.^9^

### Clinical Outcomes

To assess clinical outcomes, we evaluated radiographic union, range of motion, complications (heterotopic ossification and infection), and unplanned return to the operating room. Symptomatic implants were not considered a complication and were recorded separately. The most recently available anterior-posterior and lateral radiographs were evaluated to assess for radiographic union by the treating surgeon (fellowship-trained in orthopaedic trauma or hand/UE) and independently by the first-author (A.R.B., fifth year orthopaedic surgery resident). Range of motion was assessed by the treating surgeon for flexion contracture (ie, terminal extension), terminal flexion, and the total arc of flexion-extension at the last outpatient follow-up visit. Patients were deemed to have a functional range of motion if their flexion-extension arc was at least 30° to 130°.^19^

### Independent Variables

Detailed sociodemographic and clinical data were identified for each patient using our institutions' Enterprise Data Warehouse and the electronic medical record (Table 1). Because the patients in this study are from a similar geographic area, median income for each patient was abstracted for each patient using the ZIP code of residence based on census data.^20^ Primary health insurance was divided into three categories (private, Medicaid, and Medicare).^21^ Distal humerus fractures were classified using the AO-OTA fracture classification by the treating surgeon and independently by the first-author (A.R.B.), and patients with other fractures were classified as “multiple injuries” (binary classification).^22^ To mitigate interobserver variability during analysis, all fractures were then grouped as extra-articular (13.A) or intra-articular (partial articular [13.B] and complete articular [13.C]). The energy of injury mechanism was defined according to the Advanced Trauma Life Support guidelines.^23^ Patients who did not meet the criteria for high-energy trauma were considered low-energy trauma. Procedures were grouped as closed treatment, ORIF, or ORIF with ulnar nerve transposition (subcutaneous versus submuscular).

### Statistical Analysis

Baseline characteristics and clinical results between responders and nonresponders were compared using the Fisher exact test for categoric variables and *t*-test/analysis of variance for continuous variables to assess for response bias. Multivariable linear regression modeling was used to assess the relationship between PROs and clinical results of distal humerus fracture treatment. To adjust for factors that may confound the relationship between PROMIS PF/PROMIS UE/QuickDASH and clinical outcomes, we used forward stepwise selection to include those patients' sociodemographic and clinical variables that were notable at an alpha level of 0.10.^14^ All models were constrained to include the arc of flexion-extension and complications as relevant, independent, and noncollinear clinical outcomes. We also assessed the relationship between PROMIS PF, PROMIS UE, and QuickDASH using simple linear regression to validate our data against previous studies.^9,11,16^ *P* values <0.05 were considered statistically significant. Stata software, version 13.1 (StataCorp), was used for all analyses.

## Results

### Characteristics of Patient Population

In this cohort of 53 patients who underwent treatment of a distal humerus fracture and completed PROMs regarding UE function, most patients were women (67.9%) and Caucasian (83%). The average age was 58 years (median: 72 years; range: 22 to 94 years). Most patients carried private (56.6%) or Medicare (37.7%) insurance. The average follow-up was 10.3 ± 7.1 months. Among all injuries, 13.2% were the result of high-energy trauma, 5.7% were open, and nine patients sustained multiple fractures. Most distal humerus fractures were intra-articular (67.9%), and 84.9% of patients were treated surgically (84.9%). Approximately 45% of patients were treated by an UE specialist (hand or shoulder/elbow fellowship-trained), 58.5% of injuries were treated as inpatient procedures, and only 11.3% of patients were discharged to rehab. Responders and nonresponders were similar in almost all characteristics, except that nonresponders were younger, more likely to be on Medicare/Medicaid, and had shorter follow-up (Table 1).

### Clinical Results

After treatment, 98.1% of patients demonstrated radiographic union of their distal humerus fracture. By the final follow-up, average flexion contracture was 18°, terminal flexion was 122°, and the average arc of flexion-extension was 105°; 52.8% of patients had a functional range of motion (at least 30° to 130° flexion-extension arc). Among all patients, nine patients (14.5%) sustained at least one complication (Table 2). Four patients had heterotopic ossification, three patients had an infection, and two patients had a nonunion. Seven patients had symptomatic implants. Clinical results were similar between responders and nonresponders.

**Table 2:** Clinical Outcomes of Responders Versus Nonresponders and Patient-reported Functional Outcome of Responders

	Responders	Nonresponders	*P* Value
	No. of Patients (%) or Mean ± SD	No. of Patients (%) or Mean ± SD	
Clinical outcomes			
Radiographic union	52 (98.1)	23 (100)	0.697
Flexion contracture (degrees)	18 ± 21	19 ± 12	0.756
Terminal flexion (degrees)	122 ± 15	118 ± 16	0.331
Arc of flexion-extension	105 ± 30	99 ± 23	0.422
Functional arc of motion (30°–130°)	28 (52.8)	9 (40.9)	0.247
Complication	9 (17.0)	2 (8.7)	0.346
Unplanned return to the OR	6 (11.3)	2 (8.7)	0.732
Patient-reported functional outcomes			
PROMIS PF 10a	41.7 ± 11.1	—	
PROMIS global (physical)	44.7 ± 11.6	—	
PROMIS global (mental)	52.2 ± 10.4	—	
PROMIS UE 16a	40.8 ± 12.4	—	
QuickDASH	39.4 ± 26.5	—	

PF = physical function, PROMIS = Patient-Reported Outcomes Measurement Information System, UE = upper extremity

### Patient-reported Functional Outcome Measures

Average (±SD) PROMIS PF and UE scores were 41.7 ± 11.1 and 40.8 ± 12.4, respectively. The average QuickDASH score was 39.4 ± 26.5 (Table 2). PROMIS PF scores were associated with PROMIS UE scores (r = 0.84, *P* < 0.001) and QuickDASH scores (r = −0.55, *P* = 0.012).^11-13,16^ In addition, PROMIS UE scores were associated with QuickDASH scores (r = 0.87, *P* < 0.001).

### Association of Clinical Results With Patient-reported Outcome Measures

**Figure 1:**
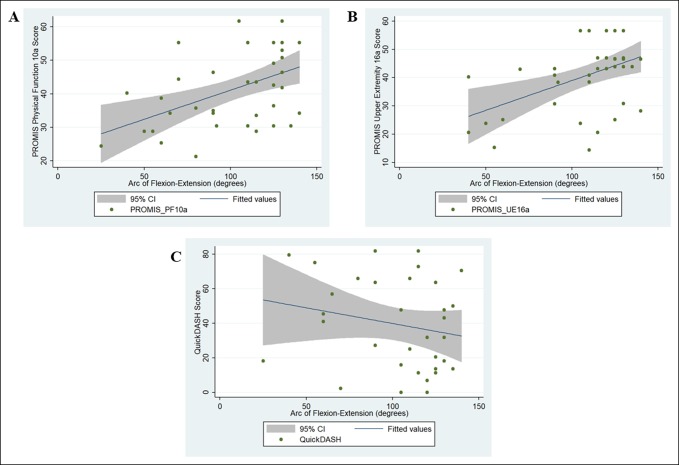
Chart showing the association between functional outcome scores and elbow range of motion (flexion-extension arc); (**A**) PROMIS PF, (**B**) PROMIS UE, and (**C**) QuickDASH. CI = confidence interval, PF = physical function, PROMIS = Patient-Reported Outcomes Measurement Information System, UE = upper extremity

**Figure 2:**
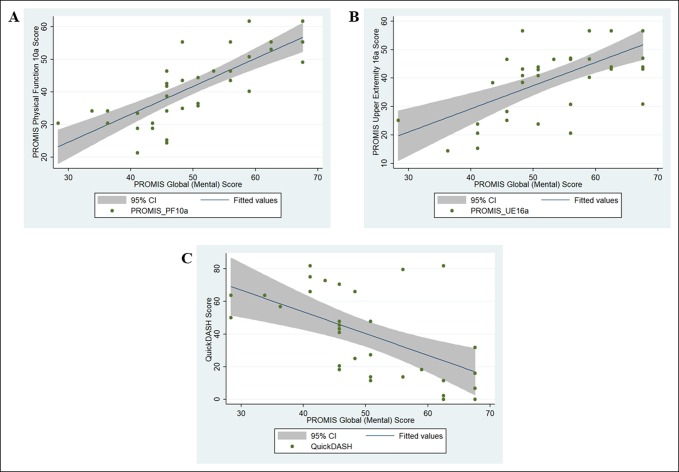
Chart showing the association between functional outcome scores and PROMIS global (mental health) subscale score; (**A**) PROMIS PF, (**B**) PROMIS UE, and (**C**) QuickDASH. CI = confidence interval, PF = physical function, PROMIS = Patient-Reported Outcomes Measurement Information System, UE = upper extremity

After controlling for likely confounding variables using multivariable analysis (eg, age and sex), the arc of flexion and extension (coefficient [95% confidence interval] = 0.13 [0.06, 0.19], *P* < 0.001) and PROMIS Global (Mental) scores (coefficient [95% confidence interval] = 0.79 [0.59, 0.99], *P* < 0.001) were independently associated with PROMIS PF scores. Similar results were observed for PROMIS UE and QuickDASH scores (Table 3, Figures 1 and 2).

**Table 3:** Multivariable Analysis of the Association Between Clinical Outcomes and Patient-reported Functional Outcomes Adjusted for Sociodemographic and Clinical Factors

	Coefficient	95% CI		*P* Value	Adjusted R^2^
PROMIS PF 10a (n = 40)					
Arc of flexion-extension	0.13	0.06	0.18	**<0.001**^a^	0.750
Complication	0.25	−4.84	5.35	0.920	
PROMIS global (mental)	0.79	0.59	0.99	**<0.001**^a^	
PROMIS UE 16a (n = 40)					
Arc of flexion-extension	0.15	0.04	0.26	**0.007**^a^	0.523
Complication	0.86	−8.09	9.81	0.847	
PROMIS global (mental)	0.73	0.38	1.09	**<0.001**^a^	
QuickDASH (n = 33)					
Arc of flexion-extension	−0.13	−0.40	0.14	0.349	0.349
Complication	−4.17	−25.2	16.9	0.688	
PROMIS global (mental)	−1.35	−2.12	−0.57	**0.001**^a^	

CI = confidence interval, DASH = Disabilities of the Arm, Shoulder, and Hand, PF = physical function, PROMIS = Patient-Reported Outcomes Measurement Information System, UE = upper extremity

## Discussion

Historically, clinical (including radiological) outcomes have been used to measure surgical treatment success and quality because they are easily obtained from administrative and clinical records, are easily quantified, and have high face validity.^24^ Yet, clinical outcomes do not capture the full patient perspective and multiple recent studies have demonstrated how PROMIS scores can be used to better describe aspects of health status that are reported directly from patients after UE trauma.^9,24^ In this study, we present data about the clinical and PROs after treatment of distal humerus fractures. Our findings demonstrate that the PROs are associated with clinical outcomes (ie, range of motion), but each of these sets of metrics has features that are unique and important when evaluating treatment effectiveness.

Although PROs capture benefits of surgical treatment beyond survival and physiologic markers, the extent to which PROs are affected by traditionally measured clinical outcomes has remained unclear, especially when using PROMIS scores, abbreviated functional outcome measures (eg, QuickDASH), or for specific clinical conditions.^24^ In this cohort of distal humerus fractures, the only clinical outcome independently associated with PROs was the arc of motion (Figure 1). On average, an increase in the arc of flexion-extension of 70° to 80° was associated with an improvement of 8 to 9 points on the PROMIS instruments.^25^ This finding is comparable to previous studies which have shown that the arc of motion was related to QuickDASH scores after elbow/wrist trauma.^15,26^ In addition, we observed that long-term outcomes (eg, final arc of motion) were more strongly associated with PROs than perioperative complications. These findings lend further support to the notion that patients are often satisfied despite adverse or unexpected events and that PROs likely reflect the durability of clinical outcomes.^14^ Our data also suggest that emphasizing efforts to improve the terminal arc of flexion-extension are likely to be associated with higher PRO. These results support a comprehensive approach to surgical quality that incorporates both clinical events and self-reported measures of health status.

We also found that the PROMIS Global (Mental) subscale was independently associated with all measures of physical or upper extremity-specific function (Figure 2). On average, increases in PROMIS Global (Mental) subscale scores of 10 to 12 points were associated with 8 to 9 point improvements on PROMIS PF or UE measures.^25^ These results are supported by multiple previous studies that have demonstrated how patient mindset may be the most important factor of self-reported outcomes.^18,27^

The importance of patient mental health in the measurement of PROs presents a plausible explanation for why PROs are not fully determined by clinical outcomes and, in part, emphasizes the importance of collecting “patient independent” outcome measures. Age, sociodemographic characteristics, or injury-related characteristics were not independently associated with PROMs in our study, although they were in others.^9,18,27^ If only PROMs are used when determining financial reimbursement, our results suggest a mechanism by which presurgery mental status may be inappropriately used to select against patients expected to have worse PROMIS PF or UE measures. This further supports the value of a physicians' judgment in the evaluation of outcomes of a care episode.^28^

This study has several limitations. There is a potential for response bias because only 69.7% of eligible patients completed an UE PROM; however, our response rate is similar to other comparable studies and patient/injury characteristics of responders and nonresponders were similar^11,16^ (Table 1). Given the retrospective nature of the study, patients had various end points of follow-up, although the effect of this is unclear. The follow-up duration was added to our regression analyses but was omitted in the final multivariable regression models because of the lack of statistically significant association. In addition, not all potential predictors could be assessed. For example, PF before the injury or other patient psychological factors (eg, PROMIS Pain Interference) may have influenced outcome measures, but these could not be retrieved retrospectively.^29^ Finally, some of the lack of influence of clinical outcomes on PROMs may be a limitation of our follow-up. We focused on shorter term PROMs in this study, but future studies should assess this in the long-term, ideally in prospective fashion, because the results may degenerate over time. Nevertheless, it is reassuring that our analysis recapitulates findings from multiple previous studies.^11-13,15,16^

## Conclusions

This study highlights the importance of measuring both clinical and PROMs when evaluating distal humerus fracture treatment effectiveness because each of these metrics is a unique assessor of outcome. Given the paucity of data regarding typical PROMIS or QuickDASH scores after distal humerus fracture treatment, our study also provides benchmark data that can be used for future comparison.^8,10^ Finally, the awareness of factors associated with poorer patient-reported and clinical outcome measures can be used to guide patient expectations and further encourage improvement in range of motion.
